# Understanding digital health literacy in the arab world: a study of arab adults with diabetes, hypertension, and rheumatoid arthritis residing in Qatar

**DOI:** 10.3389/fdgth.2026.1809014

**Published:** 2026-06-17

**Authors:** Hadeel Zaghloul, Fares Ahdab, Zahir Tag, Sanish Varghese, Batoul Arabi, Minatullah Al-Ani, Aya Abdullah, Sara Omar, Maryam Arabi, Lina Ahmed, Shadi Mahmoud, Shaza Zaghlool, Seham Alebbi, Samar Al-Emadi, Thurayya Arayssi

**Affiliations:** 1 Weill Cornell Medicine in Qatar, Education City, Qatar Foundation, Doha, Qatar; 2 Internal Medicine Department, Hamad Medical Corporation, Doha, Qatar

**Keywords:** digital health literacy, chronic disease, diabetes mellitus, hypertension, rheumatoid arthritis, patient activation, health knowledge, Arab

## Abstract

**Introduction:**

Digital health literacy (DHL)- the ability to access, understand, evaluate, and apply health information from digital sources- is increasingly essential for chronic disease self-management. Despite rapid digital health advancements in the Arab region, evidence on DHL among patients with chronic diseases remains limited. Our recent systematic review found no studies addressing DHL in this population. This study aimed to assess DHL and its associated sociodemographic and behavioral factors among Arab adults with diabetes (DM), hypertension (HTN), and rheumatoid arthritis (RA) in Qatar.

**Methods:**

A cross-sectional survey was conducted among Arab adults recruited from outpatient clinics at Hamad General Hospital, Qatar. Eligible participants were aged ≥18 years and diagnosed with DM, HTN and/or RA. The survey included the eHealth Literacy Scale (eHEALS), sociodemographic and clinical questions, digital technology use, physical activity, the Patient Activation Measure (PAM), and self-reported behavior change. Descriptive statistics, bivariate analyses, and multivariable logistic regression identified determinants of high DHL (eHEALS ≥27).

**Results:**

A total of 405 participants were included [mean age 53.8 (SD 12.6) years; 69.6% male]. The mean DHL score was 24.9 ± 7.6, with 53.1% classified as having high DHL. In multivariable analysis, participants aged ≥60 years had significantly lower odds of high DHL compared to those <45 years (aOR: 0.18; 95% CI: 0.06–0.52; *p* = 0.002), while those with an advanced degree had higher odds compared to those with a high school education or less (aOR: 3.97; 95% CI: 1.22–12.89; *p* = 0.022). Frequent internet use (several times/day: aOR: 4.21; 95% CI: 1.75–10.13; *p* = 0.001; constant use: aOR: 4.63; 95% CI: 1.75–12.26; *p* = 0.002) and use of the internet for health information (aOR: 47.71; 95% CI: 16.36–139.12; *p* < 0.001) were strong predictors of high DHL. Higher DHL was also significantly associated with greater patient activation across multiple domains (*p* < 0.05).

**Conclusions:**

DHL among Arab adults with chronic diseases in Qatar is moderate, with significant disparities by age, education, and digital engagement. Targeted interventions that translate DHL assessments into individualized self-care guidance, including simplified digital tools, accessible Arabic-language resources, and structured patient education, are needed to ensure that improved literacy translates directly into better chronic disease self-management outcomes.

## 1 Introduction

Digital health literacy (DHL), or e-Health literacy, is defined as the ability to find, understand, and evaluate health information from electronic sources and apply the knowledge gained to make health-related decisions. The term was first introduced in 2006 by Norman and Skinner, who created the eHealth literacy model outlining basic domains of skills believed to be necessary for appropriately appraising health information (1). The ability to obtain health information digitally and understand its meaning while evaluating its quality enables people to make sound health decisions (2, 3). According to the World Health Organization (WHO), digital technologies have the potential to significantly advance efforts toward achieving the Sustainable Development Goals related to health and well-being, and equity especially in underserved or resource limited settings (4). With increasing global internet connectivity and growing access to personal digital devices (5), digital health solutions offer an affordable means to engage wide populations and deliver health services at scale. In Qatar internet penetration exceeds 99% of the population reflecting near universal digital connectivity and positioning the country as a highly favourable environment for the implementation of digital health interventions (6).

Studies estimate that a substantial proportion of individuals turn to online sources for health-related information, often before consulting with a physician. Recent data indicate that approximately 50%–60% of adults report using the internet to seek health or medical information annually, with this proportion continuing to rise globally (7–9). As healthcare systems continue to undergo digital transformation, DHL has emerged as an essential skill for patient empowerment, particularly in chronic disease management. Advancements in digital health technology (DHT), including telemedicine platforms, wearable devices, mobile applications, and online health portals, are reshaping how individuals interact with the healthcare system (10, 11). These tools enable continuous monitoring, personalized feedback, remote consultations, and real-time disease management, improving patient engagement and adherence to care plans (12, 13).

Chronic diseases such as diabetes (DM), hypertension (HTN), and rheumatoid arthritis (RA) are prime candidates for digital interventions due to their long-term management needs (14). In Qatar, these conditions represent a substantial public health burden: recent estimates indicate that DM affects approximately 24.6% of the adult population, while HTN prevalence ranges from about 30% to over 40% among adults (15). RA, although less prevalent, affects approximately 0.5%–1% of adults globally and contributes significantly to chronic disability (16). These conditions require regular self-monitoring, medication adherence, and lifestyle adjustments. Numerous digital platforms are now tailored to support these conditions, such as glucose tracking apps for DM (17), blood pressure monitors for HTN (18), and online communities and educational portals for RA (19). However, patients with low DHL may struggle to navigate or trust these tools, leading to suboptimal disease management, reduced adherence, and increased healthcare utilization (20). Recent studies continue to demonstrate that low DHL remains a key barrier to the effective use of digital health technologies, contributing to reduced engagement, misunderdstanding of medical information, and suboptimal outcomes in digital interventions (20).

Qatar is a sovereign Arab state in Western Asia on the Qatar Peninsula of the larger Arabian Peninsula (21). It is a multicultural society where about 10–12 % of the total population (3.1 million) are Qatari citizens, and approximately 88–90 % are expatriates largely from South Asia, other Arab countries, and Western countries (21). Qatar has made significant investments in eHealth as part of its National Health Strategy (2011–2016), launching initiatives such as AI-powered diabetes assistants and localized mobile apps for chronic disease management (22). These efforts demonstrate the country's strong digital infrastructure and willingness to integrate DHT into mainstream healthcare. Recent data indicate substantial uptake of digital health services in Qatar, with telemedicine usage increasing by over 300% and more than 1.2 million telehealth consultations recorded in recent years. Additionally, mobile health applications have seen widespread adoption, supported by near-universal smartphone penetration (23). Yet, the true impact of these innovations depends on the population's ability to effectively use them.

Despite the global push for DHL, challenges persist. Language barriers, varying technological proficiency, cultural resistance, privacy concerns, and the spread of misinformation continue to hinder DHL uptake, particularly in the Arab world (22, 24). In particular, language remains a major barrier, as a substantial proportion of digital health content and applications are primarily available in English, limiting accessibility for Arabic-speaking populations. Even when Arabic content is available, it may vary in quality, readability, and cultural relevance, making it difficult for users to identify trustworthy sources. Additionally, cultural communication norms in the Arab world, such as preference for physician-directed care and reliance on interpersonal advice, may reduce engagement with digital platforms or affect trust in online health information (24, 25). These limitations disproportionately affect older adults, individuals with lower socioeconomic status, and those with limited education, exacerbating health disparities and undermining the promise of digital health (26).

While DHL has been extensively studied in Western and Asian populations, there remains a critical gap in research on DHL among patients with chronic diseases in Arab populations. Although several studies have explored DHL in the Middle East and North Africa (MENA) region, including among general populations and specific groups such as students and internet users in countries like Jordan, Kuwait, Lebanon, and Saudi Arabia, these studies are often limited in scope and context, frequently focusing on single-country samples or non-clinical populations (25, 27–29). Moreover, most studies do not specifically examine individuals with chronic diseases or comprehensively assess determinants of DHL in these populations. In a recent systematic review and meta-analysis conducted by our team (30), we analyzed eight studies evaluating DHL using the eHealth Literacy Scale (eHEALS) among individuals with DM, HTN, or RA. Although the pooled mean DHL score was moderately high, none of the included studies were conducted in Arab populations, and only two investigated factors associated with DHL. This gap severely limits our understanding of how cultural and contextual factors influence DHL in the MENA region.

### 1.1 Study Aim

This study aims to assess DHL and associated factors among Arab adults with DM, HTN, and RA. To provide a more comprehensive understanding of DHL, we used several validated instruments: The eHealth Literacy Scale (eHEALS) (1), to measure perceived digital health literacy, the Patient Activation Measure (PAM-12) (31) to assess participants' knowledge, skills, and confidence in managing their health (which is conceptually linked to DHL as both constructs reflect an indivdual's ability to assess, understand, and apply health information to support informed decision-making and self-management), and the International Physical Activity Questionnaire IPAQ) (32), to evaluate physical activity levels, (included as a key behavioral outcome given that higher DHL may facilitate engagement with digital health tools and information that promote healthy lifestyle behaviors such as physical activity). By integrating these tools, the study explores how DHL relates to patient activation, self-management behaviors, and physical activity levels. We also examined demographic, socioeconomic, and behavioral factors- such as age, education, income, technology use, and digital engagement- that may influence DHL. This work seeks to address a critical knowledge gap and inform strategies to enhance DHL and chronic disease management in Qatar and the broader Arab region.

## 2 Methods

### 2.1 Study design and setting

This is a cross-sectional study conducted among Arab adults residing in Qatar who were diagnosed with at least one of three chronic diseases: DM, HTN, or RA. The study was designed and carried out by researchers at Weill Cornell Medicine-Qatar (WCM-Q) and Hamad Medical Corporation (HMC), with ethical approvals obtained from the Institutional Review Boards (IRBs) of WCM-Q and HMC. Data collection took place between July 17, 2023 and February 14, 2025.

### 2.2 Participant eligibility and recruitment

Participants were eligible for inclusion if they were aged 18 years or above, resided in Qatar, self-identified as Arab by nationality or ethnicity, and had a diagnosis of at least one of the following: DM, HTN, or RA. Exclusion criteria included inability to provide informed consent or inability to complete the survey in English or Arabic. No incentive for participation was provided.

All participants were recruited using a convenience sampling approach from outpatient clinics at Hamad General Hospital, a tertiary care hospital under HMC. Eligible patients were approached in waiting areas or during clinic visits by trained bilingual (Arabic and English) research assistants. After explaining the study objectives, researchers invited patients to complete the digital survey using tablets provided by the research team via a secure Qualtrics link.

### 2.3 Justification of disease focus

Diabetes, hypertension, and rheumatoid arthritis were selected for their high burden in Qatar, their dependence on self-management, and the increasing availability of tailored digital interventions. These conditions involve continuous monitoring, lifestyle modifications, and adherence to complex care regimens, making digital health literacy a critical component of effective disease control. Additionally, our systematic review (30) found a striking lack of studies assessing DHL in Arab patients with chronic diseases, underscoring the need to explore DHL within this high-risk and culturally specific population.

### 2.4 Ethics and consent

Participation was entirely voluntary. All participants provided informed consent prior to completing the survey. The consent form was presented on the first page of the survey, detailing the purpose, eligibility criteria, confidentiality protections, potential risks, and data usage. Consent was implied by survey submission. No personally identifiable data were collected.

### 2.5 Sample size determination

A target sample size of approximately 385 participants was determined based on standard sample size calculations for cross-sectional studies estimating proportions, assuming a 95% confidence level, 5% margin of error, and a conservative expected proportion of 50% to ensure maximum variability. This sample size is considered sufficient to provide adequate statistical power to examine associations between DHL and key demographic and behavioral factors. The final sample size of 405 participants exceeded this requirement.

### 2.6 Survey instrument and measures

All survey questions were translated and culturally adapted into Arabic following standard forward–backward translation procedures to ensure linguistic and conceptual equivalence. The eHealth Literacy Scale (eHEALS), Patient Activation Measure (PAM-12), and International Physical Activity Questionnaire (IPAQ) have been previously validated in Arab populations (33–35), supporting their reliability and appropriateness for this study context. The survey comprised five structured sections and was available for participants to complete in English or Arabic:

#### 2.6.1 Digital health literacy

DHL was measured using the 8-item eHealth Literacy Scale (eHEALS). Each item is rated on a 5-point Likert scale (1 = “strongly disagree” to 5 = “strongly agree”), yielding a total score ranging from 8 to 40. (1) The total eHEALS score was calculated by summing item responses. For analysis, we dichotomized this score into “low” vs. “high” eHealth literacy groups. The cut-off was based on the sample median, an approach used in previous research when a universally established threshold for categorizing eHEALS is not available (36). In our dataset, the median score was 27, and scores ≥27 were therefore classified as indicating “high” eHEALS. This threshold is consistent with those reported in regional and international studies (29, 33). The internal consistency of the eHEALS in our sample was assessed using Cronbach's alpha, with a value above 0.70 considered acceptable (37). We also conducted an exploratory factor analysis (principal components analysis) on the eHEALS items to examine its dimensional structure, retaining factors with eigenvalues >1.0 (Kaiser's criterion) (38).

#### 2.6.2 Patient activation

The 12- item Patient Activation Measure (PAM-12) was administered to assess each participant's knowledge, skills, and confidence in managing their health (31). This instrument was included to enable an exploratory analysis of the relationship between patient activation and eHealth literacy in this population.

#### 2.6.3 Physical activity

Physical activity was evaluated using the International Physical Activity Questionnaire (IPAQ) short form (32). Following standard IPAQ scoring guidelines (39, 40), participants were categorized as having low, moderate, or high physical activity levels. For scoring purposes, metabolic equivalent (MET) values of 3.3, 4.0, and 8.0 were assigned to walking, moderate-intensity, and vigorous-intensity activities respectively (41). Total physical activity was calculated as MET-minutes per week (MET × minutes per day × days per week). Participants were classified as highly active if they reported either (1) vigorous-intensity activity on ≥3 days with ≥1500 MET-minutes per week, or (2) ≥7 days of combined walking, moderate-, or vigorous-intensity activity totaling ≥3000 MET-minutes per week. Participants were classified as moderately active if they reported either (1) vigorous-intensity activity on ≥3 days for ≥20 min per day, (2) ≥5 days of moderate-intensity activity or walking for ≥30 min per day, or (3) ≥5 days of combined activities achieving ≥600 MET-minutes per week. Participants who did not meet any of these criteria were classified as having low physical activity.

#### 2.6.4 Other predictor variables

Several predictor variables hypothesized to be associated with DHL were collected (42). Key sociodemographic characteristics such as age group, gender, nationality, and education level were recorded for each participant (42, 43). Participants' clinical profiles were documented, including their specific chronic condition diagnosis (DM, HTN, and/or RA) and the total number of these chronic conditions. Technology engagement was assessed through multiple self-reported measures. These included the frequency of internet use, the number of digital devices owned, the methods used for tracking personal health information (e.g., keeping paper records vs. using smartphone apps), use of the internet to seek health-related information, and use of any digital health-tracking tools or applications.

#### 2.6.5 Chronic disease self-management and behavioral change

Evaluated behavior changes based on digital information, communication with physicians regarding online content, and reliance on internet-based medical advice.

The estimated completion time for the survey was 20–30 min. Mandatory questions were limited to inclusion criteria and core DHL components to minimize burden.

### 2.7 Data management and analysis

Survey data were securely stored via the Qualtrics platform and exported to STATA 18 for cleaning and analysis. Descriptive statistics summarized participant characteristics and DHL levels. DHL scores were calculated by summing eHEALS responses; higher scores indicated greater perceived DHL.

Categorical variables were described as frequencies and percentages, while continuous variables (such as age and eHEALS score) were reported as means with standard deviations. Bivariate analyses were then performed to examine associations between each predictor variable and the dichotomized eHEALS outcome (high vs. low eHealth literacy). Fisher's exact test was used for categorical predictors (due to some small expected cell counts), and univariable logistic regression was used for continuous or ordinal predictors, yielding unadjusted odds ratios for the likelihood of high eHealth literacy. Variables showing a *p*-value ≤ 0.20 in the bivariate analysis were considered for inclusion in the multivariable model. A multivariable logistic regression was carried out to identify independent factors associated with high eHealth literacy. All candidate predictors with *p* ≤ 0.20 were entered, and adjusted odds ratios (aORs) with 95% confidence intervals were calculated for each variable in the final model. A two-tailed *p*-value <0.05 was considered statistically significant for the multivariable analysis. Finally, an exploratory analysis was conducted to assess the relationship between eHealth literacy and patient activation. For this, PAM-12 responses were dichotomized (e.g., into agree vs. disagree categories for each item), and chi-square tests were used to compare the proportions of high vs. low eHealth literacy across the dichotomized PAM-12 item groups.

Model diagnostics were conducted to evaluate the fit, specification, calibration, and discriminatory performance of the multivariable logistic regression models. Fit between models was assessed using the Akaike Information Criterion (AIC) and Bayesian Information Criterion (BIC), with lower values indicating better fit (44). Model specification was examined using the link test, where a correctly specified model shows a statistically significant predicted value but a non-significant squared predicted value. Calibration was assessed using the Hosmer–Lemeshow goodness-of-fit test, with a *p*-value >0.05 indicating adequate agreement between observed and predicted outcomes (45, 46). Model discrimination was evaluated using the area under the receiver operating characteristic curve (AUC), with values closer to 1 indicating a better ability to distinguish between individuals with high and low eHEALS (47).

### 2.8 Missing data handling

Missing values across the variables included in the analysis were 6.8%, which is within the commonly accepted threshold for complete case analysis (<10%) (48). Therefore, no imputation was performed.

## 3 Results

### 3.1 Descriptive statistics

A total of 405 individuals participated in the study (Table 1), with a predominance of males (69.6%), which is in line with the gender distribution in Qatar (21). The mean age of participants was 53.8 (SD12.6) years, with approximately two-thirds (66.4%) below the age of 60. Nearly half of the participants had a high school education or lower. Comorbidities were prevalent, with 45.2% reporting two conditions and 4.7% having all three conditions. Digital engagement was high, with over three-quarters of participants accessing the internet multiple times daily or constantly, and more than two-thirds using it to seek health-related information. Regarding technology adoption, 54.7% owned two or more digital devices, while 22% employed at least three different methods for health monitoring. Physical activity assessment using IPAQ revealed that most participants (58.8%) engaged in low levels of physical activity.

**Table 1:** Characteristics of study participants.

Characteristics	*N* (%*)
I. Socio-demographic factors	
Gender	
Female	123 (30.4)
Male	282 (69.6)
Age (Years)	
Less than 45	98 (24.2)
45–59	171 (42.2)
60+	136 (33.6)
Ethnicity	
Qatari	148 (36.5)
Non-Qatari	257 (63.5)
Education level	
High school or less	171 (45.8)
Bachelor	149 (40.0)
Advanced degree	53 (14.2)
Comorbidities	
Diabetes only	90 (22.2)
Hypertension only	59 (14.6)
Rheumatoid arthritis only	54 (13.3)
Two conditions	183 (45.2)
All three conditions	19 (4.7)
III. Digital connectivity and usage	
Frequency of internet use	
Once a day or less	82 (21.5)
Several times a day	156 (40.8)
Constantly	144 (37.7)
Number of digital devices owned	
1	173 (45.3)
2	130 (34.0)
3+	79 (20.7)
Number of tracking methods	
Doesn't track	103 (27.0)
1	118 (30.9)
2	77 (20.2)
3+	84 (22.0)
Uses internet for health information	
No	112 (29.6)
Yes	267 (70.5)
IV. Physical activity	
IPAQ score	
Low	220 (58.8)
Moderate	108 (28.9)
High	46 (12.3)
**Total (%)**	**405** (**100.0)**

IPAQ, international physical activity questionnaire.

* Missing values were excluded from the analysis.

(Characteristics of study participants) bold values are the total number.

### 3.2 Electronic health literacy

**Figure 1:**
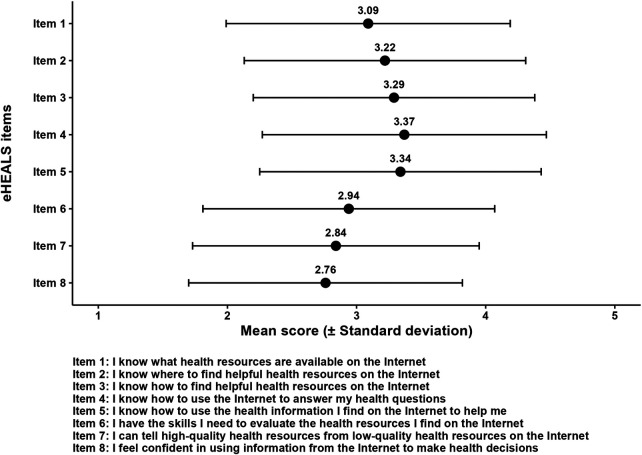
eHEALS items' mean scores.

The overall mean score across participants was 24.9 ± 7.64 (Table 2). Participants reported the highest mean score for using the internet was to answer health questions (3.37 ± 1.10) and the lowest for feeling confident in using online information to make health decisions (2.76 ± 1.06) (Figure 1).

**Table 2:** eHEALS items' mean scores and overall mean.

eHEALS questions	Mean ± SD (*N* = 384)
I know what health resources are available on the Internet	3.09 ± 1.10
I know where to find helpful health resources on the Internet	3.22 ± 1.09
I know how to find helpful health resources on the Internet	3.29 ± 1.09
I know how to use the Internet to answer my health questions	3.37 ± 1.10
I know how to use the health information I find on the Internet to help me	3.34 ± 1.09
I have the skills I need to evaluate the health resources I find on the Internet	2.94 ± 1.13
I can tell high-quality health resources from low-quality health resources on the Internet	2.84 ± 1.11
I feel confident in using information from the Internet to make health decisions	2.76 ± 1.06
Overall mean score*	24.9 ± 7.64

eHEALS, eHealth literacy scale; SD, standard deviation.

* Represents the average score across all eHEALS items.

### 3.3 Reliability of the score

The eHEALS demonstrated strong internal consistency, with a Cronbach's alpha of 0.95 (Appendix A). Removing any individual item had minimal impact on reliability, as item-specific Cronbach's alpha values ranged from 0.94 to 0.96. Factor analysis revealed a dominant first factor with an eigenvalue of 6.1, accounting for 75.8% of the variance, while the second factor had a markedly lower eigenvalue of 0.6, contributing only 8.1%. All items loaded strongly on the first factor (≥0.5), with factor loadings ranging from 0.66 to 0.92. The sharp decline in eigenvalues supports a single underlying construct.

### 3.4 High eHEALS percentages

Overall, 53.1% of participants demonstrated high eHEALS (95% CI: 48.0–58.2%) (Table 3). High eHEALS was more prevalent among younger participants, with the highest percentage observed in those under 45 years, gradually decreasing with increasing age. When examining conditions, those with only diabetes had the highest percentage (72.0%), while those with all three conditions had the lowest (35.3%). Participants who used the internet for health information showed considerably higher eHEALS (73.4%) compared to non-users (5.4%). Additionally, Higher eHEALS percentages were observed among participants with higher degrees, increased internet use, greater device ownership, more frequent health tracking, and higher levels of physical activity.

**Table 3:** Associations of characteristics with eHEALS.

Characteristics	Total	High eHEALS	
	*N* (%*)	^N^ (%*)	*p*-value
I. Socio-demographic factors			
Gender			
Female	115 (30.0)	53 (46.1)	0.075
Male	269 (70.1)	151 (56.1)	
Age (Years)			
Less than 45	86 (22.4)	63 (73.3)	<0.001
45–59	169 (44.0)	104 (61.5)	
60+	129 (33.6)	37 (28.7)	
Ethnicity			
Qatari	140 (36.5)	58 (41.4)	0.001
Non-Qatari	244 (63.5)	146 (59.8)	
Education level			
High school or less	170 (46.0)	61 (35.9)	<0.001
Bachelor	147 (39.7)	100 (68.0)	
Advanced degree	53 (14.3)	40 (75.5)	
Comorbidities			
Diabetes only	82 (21.4)	59 (72.0)	0.002
Hypertension only	56 (14.6)	26 (46.4)	
Rheumatoid arthritis only	51 (13.3)	26 (51.0)	
Two conditions	178 (46.4)	87 (48.9)	
All three conditions	17 (4.4)	6 (35.3)	
III. Digital connectivity and usage			
Frequency of internet use			
Once a day or less	82 (21.5)	19 (23.2)	<0.001
Several times a day	156 (40.9)	91 (58.3)	
Constantly	143 (37.5)	94 (65.7)	
Number of digital devices owned			
1	172 (45.1)	54 (31.4)	<0.001
2	130 (34.1)	90 (69.2)	
3+	79 (20.7)	60 (76.0)	
Number of tracking methods			
Doesn't track	103 (27.0)	39 (37.9)	<0.001
1	117 (30.7)	54 (46.2)	
2	77 (20.2)	53 (68.8)	
3+	84 (22.1)	57 (67.9)	
Uses internet for health information			
No	112 (29.6)	6 (5.4)	<0.001
Yes	267 (70.5)	196 (73.4)	
IV. Physical activity			
IPAQ score			
Low	220 (58.8)	109 (49.6)	0.054
Moderate	108 (28.9)	63 (58.3)	
High	46 (12.3)	31 (67.4)	
**Total**	384* **(100.0)**	**204** **(53.1)**	**NA**

eHEALS, eHealth literacy scale; IPAQ, international physical activity questionnaire.

* Missing values were excluded from the analysis. *N* = 384 reflects the sample after excluding participants with missing values for the outcome.

### 3.5 Associations with high eHEALS

Table 4 presents the results of the multivariable logistic regression analysis. Participants with an advanced degree showed significantly higher eHEALS odds- approximately four times higher-than those with a high school degree or less (aOR: 3.97, 95% CI: 1.22–12.89). Those aged over 60 years had 82% lower odds of achieving a high eHEALS compared to those under 45 years (aOR: 0.18, 95% CI: 0.06–0.52). Additionally, those diagnosed solely with rheumatoid arthritis had significantly lower odds of high eHEALS compared to those with diabetes only (aOR: 0.30, 95% CI: 0.09–0.95). No significant associations with high eHEALS were found for gender and ethnicity.

**Table 4:** Factors associated with high eHEALS.

Characteristics	Univariable regression analysis			Multivariable regression analysis	
	OR (95% CI)	*p*-value	LR test *p*-value*	aOR (95% CI)	*p*-value^†^
I. Socio-demographic factors					
Gender					
Female	1.00		0.071	1.00	
Male	1.50 (0.97–2.32)	0.071		0.67 (0.31–1.45)	0.306
Age (Years)					
Less than 45	1.00		<0.001	1.00	
45–59	0.58 (0.33–1.03)	0.064		0.42 (0.16–1.06)	0.065
60+	0.15 (0.08–0.27)	<0.001		0.18 (0.06–0.52)	0.002
Ethnicity					
Qatari	1.00		0.001	1.00	
Non-Qatari	2.11 (1.38–3.21)	0.001		1.28 (0.65–2.51)	0.476
Education level					
High school or less	1.00		0.002	1.00	
Bachelor	4.24 (1.40–12.84)	0.011		1.47 (0.70–3.11)	0.313
Advanced degree	13.30 (4.38–40.39)	<0.001		3.97 (1.22–12.89)	0.022
Comorbidities					
Diabetes only	1.00		0.002	1.00	
Hypertension only	0.34 (0.17–0.69)	0.003		0.63 (0.21–1.94)	0.422
Rheumatoid arthritis only	0.41 (0.20–0.84)	0.015		0.30 (0.09–0.95)	0.041
Two conditions	0.37 (0.21–0.66)	0.001		0.84 (0.31–2.29)	0.739
All three conditions	0.21 (0.07–0.64)	0.006		0.79 (0.10–6.48)	0.826
III. Digital connectivity and usage					
Frequency of internet use					
Once a day or less	1.00		<0.001	1.00	
Several times a day	3.67 (0.77–17.48)	0.103		4.21 (1.75–10.13)	0.001
Constantly	13.30 (2.99–59.10)	0.001		4.63 (1.75–12.26)	0.002
Number of digital devices owned					
1	1.00		<0.001	1.00	
2	4.92 (3.00–8.04)	<0.001		1.19 (0.55–2.54)	0.660
3+	5.93 (2.97–11.85)	<0.001		2.00 (0.78–5.17)	0.150
Number of tracking methods					
Doesn't track	1.00		<0.001	1.00	
1	1.41 (0.82–2.41)	0.215		0.97 (0.39–2.42)	0.949
2	3.62 (1.94–6.77)	<0.001		1.00 (0.33–2.99)	0.994
3+	3.46 (1.89–6.36)	<0.001		1.78 (0.51–6.14)	0.364
Uses internet for health information					
No	1.00		<0.001	1.00	
Yes	48.77 (20.51–115.96)	<0.001		47.71 (16.36–139.12)	<0.001
IV. Physical activity					
IPAQ score					
Low	1.00		0.050	1.00	
Moderate	1.43 (0.90–2.27)	0.135		1.48 (0.69–3.21)	0.316
High	2.10 (1.08–4.12)	0.030		2.12 (0.75–6.00)	0.156

aOR, adjusted odds ratio; CI, confidence interval; eHEALS, eHealth literacy scale; IPAQ, international physical activity questionnaire; LR, likelihood ratio; OR, odds ratio.

* Covariates with *p*-value ≤0.2 in the univariable analysis were included in the multivariable analysis.

† Covariates with *p*-value <0.05 in the multivariable analysis were considered as showing statistically significant evidence for an association with high eHEALS.

**Figure 2:**
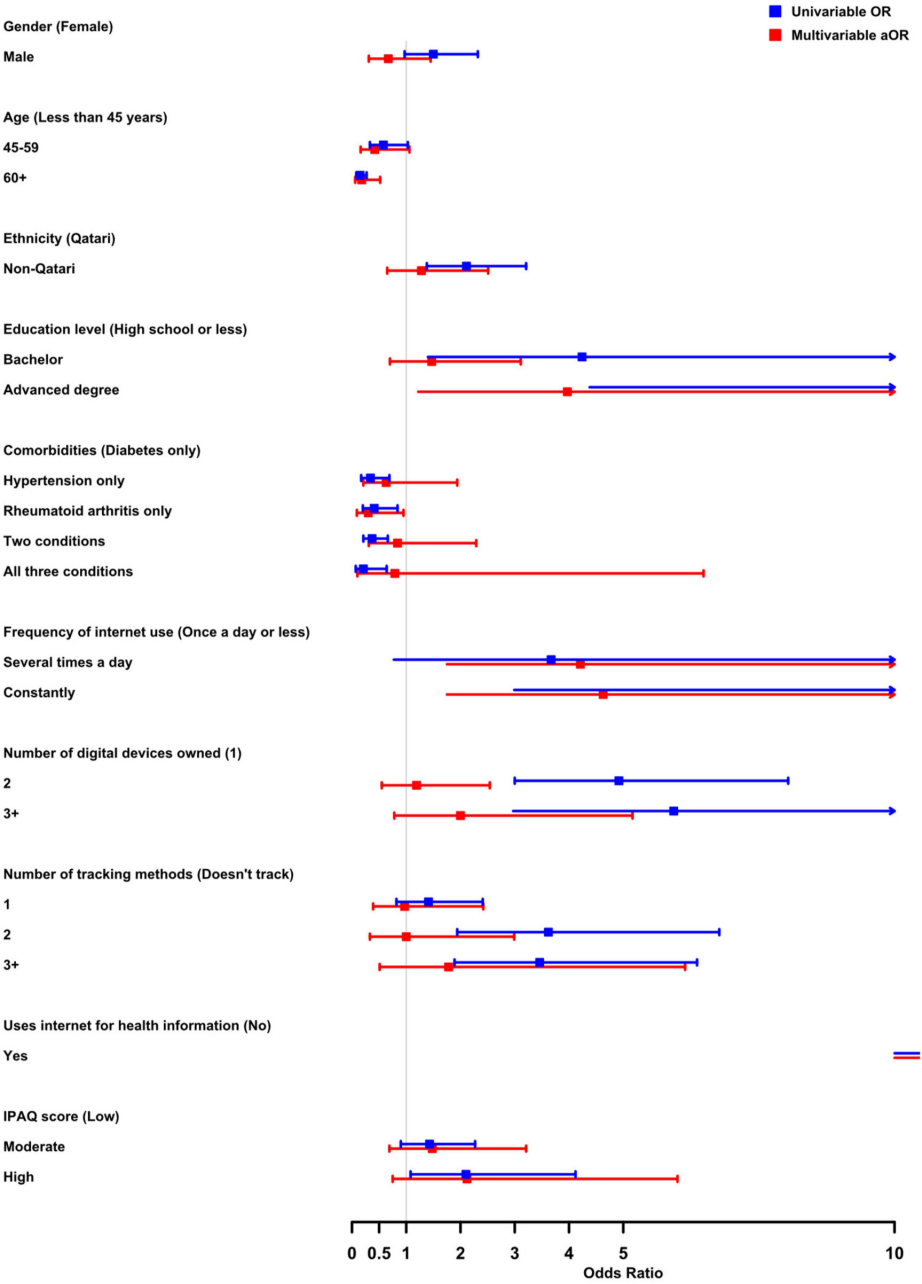
Forest plot of odds ratios of variables having associations with high eHEALS in univariable and multivariable analysis.

The odds of high eHEALS were significantly higher among frequent internet users compared to those who used the internet once a day or less, with aOR of 4.21 (95% CI: 1.75–10.13) for several times a day, and 4.63 (95% CI: 1.75–12.26) for constant usage. Similarly, participants who used the internet specifically for health information had substantially higher eHEALS odds compared to non-users (aOR: 47.71, 95% CI: 16.36–139.12). Although not statistically significant, trends showed that participants with higher levels of physical activity and greater device ownership had higher eHEALS odds compared to those with low levels of physical activity and ownership of only one device (Figure 2).

### 3.6 Model diagnostics

The full multivariable model demonstrated strong diagnostic performance (Table 5). Model fit statistics indicated good fit (AIC = 304.3; BIC = 390.2), and the link test supported correct model specification, with a statistically significant predicted value and a non-significant squared predicted value. The Hosmer-Lemeshow test further indicated adequate model calibration. In addition, the model demonstrated high discriminatory ability (AUC = 0.898). In comparison, the model excluding “uses internet for health information” showed poorer fit and discrimination, with higher AIC and BIC values and lower discriminatory ability (AUC = 0.836).

**Table 5:** Comparison of model fit and performance diagnostics across models.

Diagnostics	Full multivariable model	Full multivariable model excluding “uses internet for health information”
Log-likelihood	−131.2	−175.8
AIC*	304.3	391.6
BIC*	390.2	467.8
Predicted value *p*-value^†^	<0.001	<0.001
Squared predicted value *p*-value^†^	0.377	0.782
Hosmer–Lemeshow test *p*-value^‡^	0.100	0.265
AUC^§^	0.898	0.836

AIC, akaike information criterion; AIC, akaike information criterion; BIC, bayesian information criterion; AUC, area under the receiver operating characteristic curve.

* A lower criterion indicates a better model fit.

† Assesses model specification in logistic regression. In a correctly specified model, the predicted value should be statistically significant, whereas the squared predicted value should not be statistically significant.

‡ Evaluates the agreement between observed and predicted probabilities. A *p*-value greater than 0.05 indicates no evidence of poor model fit and suggests adequate model calibration.

§ Evaluates the model's discrimination ability, reflecting how well the model distinguishes between individuals with and without the outcome. AUC values range from 0.5 (no discrimination) to 1.0 (perfect discrimination), with higher values indicating better discriminatory performance.

### 3.7 Association of PAM with eHEALS

The results indicate a strong association between most PAM items and high eHEALS (Table 6). Participants who acknowledged personal responsibility for their health and emphasized active engagement in healthcare showed significantly higher percentages of high eHEALS. Similarly, confidence in preventing or reducing health problems, knowing how to prevent health problems, and understanding prescribed medications, health conditions, and available treatments was positively associated with high eHEALS. While confidence in following through with medical treatments at home was significantly associated with high eHEALS, the ability to communicate concerns to a doctor, even when not asked, did not show a significant relationship. Additionally, maintaining lifestyle changes, such as diet and exercise, was strongly associated with high eHEALS, whereas the ability to sustain these changes during stressful periods was not. Problem-solving ability in health management also emerged as having a strong association with high eHEALS.

**Table 6:** Association between patient activation measure items and high eHEALS.

Items	Total	High eHEALS	
	*N* (%)	*N* (%)	*p*-value
When all is said and done, I am the person who is responsible for taking care of my health			
Disagree	25 (6.7)	2 (8.0)	<0.001
Agree	347 (93.3)	200 (57.6)	
Taking an active role in my own health care is the most important thing that affects my health			
Disagree	14 (3.9)	1 (7.1)	<0.001
Agree	350 (96.2)	200 (57.1)	
I am confident that I can help prevent or reduce problems associated with my health			
Disagree	23 (6.4)	2 (8.7)	<0.001
Agree	337 (93.6)	197 (58.5)	
I know what each of my prescribed medications do			
Disagree	16 (4.4)	4 (25.0)	0.021
Agree	352 (95.7)	195 (55.4)	
I am confident that I can tell a doctor concerns I have even when he or she does not ask			
Disagree	10 (2.7)	3 (30.0)	0.196
Agree	359 (97.3)	197 (54.9)	
I am confident that I can follow through on medical treatments I may need at home			
Disagree	5 (1.4)	0 (0.0)	0.020
Agree	364 (98.6)	201 (55.2)	
I understand my health problems and what causes them			
Disagree	15 (4.0)	3 (20.0)	0.008
Agree	357 (96.0)	199 (55.7)	
I know what treatments are available for my health problems			
Disagree	18 (5.1)	3 (16.7)	0.001
Agree	335 (94.9)	192 (57.3)	
I have been able to maintain lifestyle changes, like eating right or exercising			
Disagree	80 (22.7)	29 (36.3)	<0.001
Agree	273 (77.3)	165 (60.4)	
I know how to prevent problems with my health			
Disagree	21 (5.8)	5 (23.8)	0.006
Agree	344 (94.3)	194 (56.4)	
I am confident I can figure out solutions when new problems arise with my health			
Disagree	39 (11.4)	8 (20.5)	<0.001
Agree	304 (88.6)	184 (60.5)	
I am confident that I can maintain lifestyle changes, like eating right and exercising, even during times of stress			
Disagree	86 (24.4)	42 (48.8)	0.262
Agree	266 (75.6)	150 (56.4)	
Total	384 (100.0)	204 (53.1)	NA

eHEALS, eHealth Literacy Scale.

## 4 Discussion

This study provides critical insight into the level and determinants of DHL among Arab adults with DM, HTN, and RA in Qatar. The findings reveal both encouraging trends and persistent challenges, underscoring the urgent need to tailor digital health interventions to vulnerable populations within the Arab region.

### 4.1 Key findings and interpretation

The overall DHL score, measured using the validated eHEALS tool, was moderate, with only approximately half of participants classified as having high DHL. Compared to our findings, a multinational survey conducted across four high-income European countries (United Kingdom, Sweden, Italy, and Germany) reported higher overall DHL scores (49). This cross-sectional online study, which included more than 6,000 participants and also used the eHEALS to assess DHL, found a mean eHEALS score of 29.2, substantially higher than in our cohort. This difference likely reflects the advantages of greater digital infrastructure, widespread technology adoption, and higher baseline digital engagement in these settings compared to our study population. It is also worth noting that Qatar carries a substantially higher burden of the chronic diseases studied here compared to these European countries. The MENA region has the world's highest age-standardised diabetes prevalence at 19.9%, compared to 9.8% for the IDF Europe region (50), and Qatar specifically has been ranked among the top 5 countries globally for diabetes prevalence (15). Hypertension affects approximately 32%–33% of Qatari adults (15), while high-income European countries - including the UK, Germany, and Sweden- have seen markedly lower and declining rates over recent decades (51). This higher disease burden, combined with lower DHL scores, underscores the particular urgency of addressing digital health literacy gaps in this population, where the potential consequences of inadequate self-management are disproportionately large.

Our findings are also broadly consistent with studies conducted in Arab populations, although important contextual differences exist. For example, a study from Kuwait (25) reported a mean eHEALS score of 28.6 among internet users, while a study in Jordan found similar scores among university students (29). Likewise, research conducted in Lebanon among adult internet users demonstrated moderate levels of eHealth literacy, comparable to those observed in our study (27). However, these studies primarily focused on younger, more educated, or general population samples, which may explain their relatively higher scores compared to our cohort of patients with chronic diseases. In contrast, our findings provide more clinically relevant insights by focusing specifically on individuals managing chronic conditions, who may face additional cognitive, behavioral, and health system-related barriers to effectively utilizing digital health information.

Our study found the lowest scoring item was participants' confidence in using online information to make health decisions, highlighting a major gap in the capacity to critically appraise and act on digital health content. This finding aligns with previous studies that identify confidence and critical appraisal as key barriers to effective use of digital health tools, particularly among older adults and those with less formal education (1).

Encouragingly, DHL was significantly higher among younger individuals, those with higher education, frequent internet users, and those who actively seek health information online. These trends are consistent with global research emphasizing that younger, more educated, and digitally connected populations demonstrate better navigation of online health resources (20, 49). Of particular note is the finding that participants who used the internet for health information were almost 48 times more likely to exhibit high DHL. This association may reflect a reinforcing cycle in which actively seeking health information online builds familiarity with digital health resources and, over time, sharpens the skills needed to locate, evaluate, and apply them - a pattern consistent with prior studies reporting positive associations between online health information seeking and higher eHEALS among healthcare professionals and general adult populations (52, 53). Additionally, individuals diagnosed only with DM had the highest DHL scores, possibly due to the wide availability of digital tools and education programs tailored specifically for diabetes management. In contrast, participants with multiple chronic conditions or RA reported significantly lower DHL. This is also in line with findings from our systematic review that demonstrated that patients with RA had lower DHL than those with other chronic conditions (30). This discrepancy may reflect both the cognitive and emotional burden of managing multiple conditions, as well as a lack of condition-specific digital resources in Arabic or regionally relevant content.

### 4.2 Sociodemographic and behavioral predictors

The multivariable analysis further confirms that education level, age, and digital engagement are independent predictors of high DHL. Those aged 60 and above were significantly less likely to have high DHL, reaffirming concerns about the digital divide among older populations (20). Conversely, participants with advanced degrees were nearly four times more likely to demonstrate high DHL than those with a high school education or less. Our findings align with those of a recent systematic review, which synthesized evidence from 36 studies examining sociodemographic determinants of DHL. Using meta-analyses for age and sex, the review similarly reported that older age negatively impacts DHL, while higher education levels and greater digital access are associated with improved literacy. This convergence of evidence reinforces the role of structural factors- age, education, and digital engagement-as key determinants of DHL (54).

Although we did not find a statistically significant relationship between device ownership and physical activity with DHL in the multivariable model, we observed a positive trend. This warrants further exploration, particularly in the context of health promotion initiatives that integrate wearable devices or mobile applications into lifestyle interventions.

### 4.3 DHL and patient activation

The strong association between high DHL and most items on the Patient Activation Measure (PAM) further substantiates the notion that DHL is not only a technical skill but a key driver of self-efficacy, engagement, and proactive health behaviors. Participants with high DHL were significantly more likely to agree with statements indicating responsibility for their health, confidence in self-care, and knowledge of their medications and treatment options. These findings echo literature suggesting that eHealth literacy plays a pivotal role in fostering empowered, activated patients who can navigate complex health systems more effectively (31). Several mechanisms may explain the observed relationship between DHL and patient activation. Individuals with higher DHL are better able to access, interpret, and critically evaluate digital health information, which can enhance their understanding of disease processes and treatment options. This improved knowledge base may increase confidence in making health-related decisions and foster a greater sense of control over one's health. Additionally, higher DHL may facilitate more effective use of digital tools, such as patient portals, mobile health applications, and online educational resources, which support self-monitoring, adherence to treatment, and proactive engagement with healthcare services. Collectively, these pathways may contribute to higher levels of patient activation by reinforcing both the cognitive and behavioral components of self-management.

Interestingly, the ability to communicate concerns to physicians and maintain lifestyle changes during stress were not significantly associated with DHL. This lack of association may be explained by the multifactorial nature of these behaviors, which extend beyond DHL. For example, communication with physicians may be influenced more strongly by cultural norms, health system dynamics, and patient-provider relationships than by an individual's ability to access or interpret digital health information. Similarly, the ability to maintain lifestyle changes during periods of stress may depend on psychological resilience, social support, and environmental factors, which are not directly captured by DHL. These findings suggest that while DHL is an important component of patient activation, it does not fully account for all dimensions of self-management, highlighting the need for integrated interventions that address both digital skills and broader behavioral and psychosocial determinants.

### 4.4 Implications for Qatar and the Arab Region

This study fills an important gap in the literature by focusing on DHL in the Arab world, where evidence remains limited despite widespread digital health investments. Qatar's e-Health initiatives (22) have been promising, especially in diabetes and obesity management, yet this study reveals that digital uptake alone is insufficient without parallel efforts to build user capacity, health comprehension, and trust.

Low DHL, if unaddressed, risks exacerbating health disparities and undermining the potential of digital health to promote equity. Cultural sensitivities, language barriers, and fears around privacy and misinformation continue to hinder the effective use of digital tools. For example, individuals uncomfortable with English-language platforms or skeptical of app-based advice may refrain from engaging with potentially beneficial resources. Although linguistic and cultural factors were not directly measured in this study, they likely influence DHL in this population. In particular, reliance on Arabic-language resources, variability in the quality of translated digital health content, and cultural preferences for physician-led care may shape how individuals access, interpret, and trust digital health information.

The findings support the need for targeted DHL interventions, particularly among older adults, individuals with multiple comorbidities, and those with limited education. Programs should focus on simplifying digital interfaces, offering multilingual content (particularly in Arabic), and integrating DHL training into chronic disease management programs. Interventions should also be culturally sensitive, taking into account preferences for physician-led care, the role of family in health decision-making, and varying levels of trust in digital sources. Tailoring content to align with cultural values and communication norms may improve engagement and uptake of digital health tools. Digital health tools should be designed with usability in mind, including simple interfaces, clear navigation, and content adapted to different literacy levels. Features such as visual aids, step-by-step guidance, and language customization can enhance accessibility, particularly for older adults and individuals with lower digital proficiency. Additionally, healthcare providers should be trained to assess patients' DHL levels and provide tailored support, such as recommending credible online resources or demonstrating the use of digital tools during clinic visits.

The finding that nearly half of participants had two or more chronic conditions, and that multi-morbidity was associated with lower DHL, highlights a critical gap in current digital health offerings. Most existing tools are condition-siloed, glucose tracking apps for diabetes, blood pressure monitors for hypertension, yet patients managing multiple diseases face compounded self-care demands that such tools may not adequately address. Integrated digital platforms that consolidate shared health education elements (physical activity, dietary guidance, medication adherence) while retaining condition-specific modules are therefore needed. Such platforms should be accompanied by structured clinician-guided instruction, particularly for older adults and those with lower baseline DHL, and must prioritize usability and cultural appropriateness, including Arabic-language interfaces and simplified navigation, to ensure equitable engagement among multi-morbid patients in this population.

While this study was conducted in Qatar, the findings may have broader relevance to other countries in the MENA region, particularly those with similar cultural, linguistic, and demographic characteristics. Factors such as age, education level, and digital engagement, which were identified as key determinants of DHL in this study, have also been reported in other regional and global studies, suggesting potential generalizability of these associations (3, 49). However, caution is warranted when extrapolating these findings across the MENA region, as countries differ in terms of healthcare infrastructure, digital health maturity, internet accessibility, and socioeconomic conditions. Therefore, while our findings provide valuable insights, further research across diverse MENA settings is needed to better understand context-specific determinants of DHL and to inform tailored interventions.

Although improving DHL is an important objective, the ultimate goal is to enhance patients' self-care abilities. DHL assessment should therefore serve as a diagnostic tool to stratify patients by their capacity to engage with digital health resources, and to tailor the guidance they receive accordingly. For example, patients with low DHL, particularly older adults and those with lower education, may benefit most from direct, face-to-face instruction in how to use specific digital tools, supported by simplified Arabic-language materials. In contrast, patients with high DHL who are already active online health-information seekers may benefit from guided curation of high-quality, evidence-based digital resources. Integrating DHL screening into routine chronic disease consultations could allow clinicians to match patients with the level and type of digital support most likely to improve their self-management behaviors.

From a policy perspective, these findings highlight the need to integrate digital DHL into Qatar's national digital health strategy. This includes incorporating DHL assessment into routine care, developing culturally appropriate Arabic-language digital resources, and training healthcare providers to support patients in using digital health tools. Such efforts are essential to ensure equitable access and maximize the impact of digital health initiatives.

### 4.5 Strengths and limitations

A major strength of this study is its focus on a high-risk, understudied population in a rapidly digitizing healthcare environment. The use of a validated DHL measure and linkage with behavioral and demographic variables allows for robust conclusions. However, several limitations should be considered. The cross-sectional design precludes causal inference, and the reliance on self-reported measures of technology use and health behaviors may introduce response bias. Moreover, the study was conducted at a tertiary care center using clinic-based recruitment, which may limit generalizability to the broader population, particularly rural or under-resourced groups. This approach may also introduce selection bias, as individuals attending outpatient clinics are more likely to be engaged with healthcare services and may have higher health awareness and access to information, potentially leading to an overestimation of digital health literacy levels. In addition, the sample was predominantly male, reflecting local demographic patterns in Qatar; however, this imbalance may limit generalizability, particularly with respect to gender differences in digital engagement and health information-seeking behaviors. Furthermore, key socioeconomic variables, such as income, employment type, marital status, and affordability of digital access, were not comprehensively assessed. These factors are known to influence DHL, and their omission may result in residual confounding and limit a more nuanced understanding of its determinants. Finally, the PAM-12 activation score could not be calculated because the validated scoring algorithm is proprietary and not publicly available without licensing. Instead, individual PAM-12 items were dichotomized and analyzed in relation to eHEALS as part of an exploratory analysis.

### 4.6 Future directions

Future research should explore longitudinal outcomes to assess whether improvements in DHL lead to better health outcomes over time. There is a need to integrate DHL into national health strategies to ensure that digital transformation efforts are aligned with population needs and capacities. Longitudinal studies are particularly important to establish causal relationships between DHL, patient activation, and health outcomes, and to evaluate the sustained impact of targeted interventions over time.There is also a need to evaluate specific interventions, such as digital skills training, app-based coaching, or culturally tailored content, and their effectiveness in raising DHL and empowering chronic disease patients. Finally, policymakers and health system leaders should consider incorporating DHL as a key performance indicator in national digital health strategies.

## Data Availability

The raw data supporting the conclusions of this article will be made available by the authors, without undue reservation.

## Appendix A. Reliability statistics of eHEALS.

**Table:**

eHEALS question	Cronbach's alpha if item removed	First factor	Second factor
I know what health resources are available on the Internet	0.95	0.88	−0.04
I know where to find helpful health resources on the Internet	0.94	0.92	−0.24
I know how to find helpful health resources on the Internet	0.95	0.89	−0.23
I know how to use the Internet to answer my health questions	0.94	0.90	−0.14
I know how to use the health information I find on the Internet to help me	0.94	0.89	−0.07
I have the skills I need to evaluate the health resources I find on the Internet	0.95	0.84	0.31
I can tell high-quality health resources from low-quality health resources on the Internet	0.95	0.83	0.33
I feel confident in using information from the Internet to make health decisions	0.96	0.66	0.18
Overall Cronbach's alpha	0.95	−	−
Eigenvalue	−	6.1	0.6
Variance accounted (%)	−	75.8	8.1

eHEALS, eHealth literacy scale.
